# Is It Possible to Detect Activated Brown Adipose Tissue in Humans Using Single-Time-Point Infrared Thermography under Thermoneutral Conditions? Impact of BMI and Subcutaneous Adipose Tissue Thickness

**DOI:** 10.1371/journal.pone.0151152

**Published:** 2016-03-11

**Authors:** Sergios Gatidis, Holger Schmidt, Christina A. Pfannenberg, Konstantin Nikolaou, Fritz Schick, Nina F. Schwenzer

**Affiliations:** 1 University of Tübingen, Department of Radiology, Diagnostic and Interventional Radiology, Tübingen, Germany; 2 University of Tübingen, Department of Radiology, Section for Experimental Radiology, Tübingen, Germany; University of Minnesota, UNITED STATES

## Abstract

**Purpose:**

To evaluate the feasibility to detect activated brown adipose tissue (BAT) using single-time-point infrared thermography of the supraclavicular skin region under thermoneutral conditions. To this end, infrared thermography was compared with 18-F-FDG PET, the current reference standard for the detection of activated BAT.

**Methods:**

120 patients were enrolled in this study. After exclusion of 18 patients, 102 patients (44 female, 58 male, mean age 58±17 years) were included for final analysis. All patients underwent a clinically indicated 18F-FDG-PET/CT examination. Immediately prior to tracer injection skin temperatures of the supraclavicular, presternal and jugular regions were measured using spatially resolved infrared thermography at room temperature. The presence of activated BAT was determined in PET by typical FDG uptake within the supraclavicular adipose tissue compartments. Local thickness of supraclavicular subcutaneous adipose tissue (SCAT) was measured on CT. Measured skin temperatures were statistically correlated with the presence of activated BAT and anthropometric data.

**Results:**

Activated BAT was detected in 9 of 102 patients (8.8%). Local skin temperature of the supraclavicular region was significantly higher in individuals with active BAT compared to individuals without active BAT. However, after statistical correction for the influence of BMI, no predictive value of activated BAT on skin temperature of the supraclavicular region could be observed. Supraclavicular skin temperature was significantly negatively correlated with supraclavicular SCAT thickness.

**Conclusion:**

We conclude that supraclavicular SCAT thickness influences supraclavicular skin temperature and thus makes a specific detection of activated BAT using single-time-point thermography difficult. Further studies are necessary to evaluate the possibility of BAT detection using alternative thermographic methods, e.g. dynamic thermography or MR-based thermometry taking into account BMI as a confounding factor.

## Introduction

With the introduction of 18F-FDG PET/CT it became clear that brown adipose tissue (BAT) is prevalent not only in children but also in a significant proportion of adults [1–3]. Cold exposure has been shown to be a major trigger of BAT activation [4]. Under thermoneutral conditions, hyperthyreoidism and psychological stress have been reported to result in BAT activation [5,6].

Clinical studies in patients that were examined by 18F-FDG PET/CT revealed demographic factors associated with a higher prevalence of active BAT under thermoneutral conditions, namely young age, low body-mass index (BMI) and female sex [7]. These results led to the assumption that BAT is involved as a protective factor in the pathogenesis of metabolic disorders including diabetes and obesity [8]. This assumption is supported by animal studies [9,10] and by retrospective demographic studies in humans [7,11,12].

Despite these results, many aspects of BAT physiology in humans remain unclear. This is mainly due to the challenge of BAT detection in humans. The current gold standard for the detection of activated BAT is 18F-FDG PET. Metabolically active BAT, mainly located in the supraclavicular fat compartments, shows a high glucose uptake which can be visualized using 18F-FDG-PET [1]. However, PET is associated with radiation exposure and high cost. The application of PET in healthy volunteers is thus limited. Chemical shift MRI is being discussed as a non-invasive alternative to FDG-PET for the detection of BAT [13]. However, detection of BAT by MRI relies on morphological features and does not allow for discrimination between active and inactive BAT [14].

Infrared thermography was proposed as a further alternative for the detection of brown adipose tissue. A central property of brown adipose tissue is the generation of heat [15]. Infrared thermography as a non-invasive, simple-to-use and inexpensive technique might thus allow for an efficient detection of metabolically active BAT. This technique has already been found to correlate well with 18F-FDG uptake of BAT in mice [16]. First case reports and feasibility studies in humans hypothesized an increase in local skin temperature in the supraclavicular region by activation of underlying brown adipose tissue [17–19]. Recent studies compared dynamic infrared thermography with FDG-PET in individuals before and after cold exposure showing the feasibility of BAT detection using infrared thermography in this experimental setting [20,21]. However, these studies did not evaluate the feasibility of detecting activated BAT under thermoneutral conditions using single-time-point thermography and did not correct the observed results for confounding factors, especially the body mass index. The possibility of detecting activated BAT under thermoneutral conditions would potentially give insight into mechanisms of BAT activation beyond cold exposure, e.g. nutritional, hormonal or life-style factors.

The aim of this study was to evaluate the feasibility to detect activated brown adipose tissue (BAT) using single-time-point infrared thermography of the supraclavicular skin region under thermoneutral conditions. To this end, infrared thermography was compared with 18-F-FDG PET, the current reference standard for the detection of activated BAT.

## Materials and Methods

### Patients

This study was approved by the Ethics Committee of the University of Tübingen. 120 patients (mean age 58±16 years, range 18–85 years, 54 female) were prospectively enrolled from October 2013 to June 2014. All patients gave their written informed consent. Inclusion criteria were: clinically indicated 18-F-FDG PET/CT including the neck region and age ≥ 18 years. Exclusion criteria were: tumor or skin disease in the neck region and fever.

Of the 120 patients enrolled in this study, 18 were excluded from analysis due to tumors of the neck region detected in PET/CT (n = 8) and insufficient image quality of infrared thermography (n = 10). 102 patients were finally included in the final analysis. Patient characteristics are summarized in Table 1.

**Table 1:** Patient characteristics.

	all patients (n = 102)	BAT—(n = 93)	BAT + (n = 9)	
**age [years]**	58±17	61±14	31±14	p<0.001
**BMI [kg/m**^**2**^**]**	26±5	27±5	23±5	p = 0.01
**body weight [kg]**	78±18	79±18	71±22	
**height [cm]**	173±11	172±11	176±13	
**% female**	43	42	56	

BAT +: patients with activated BAT detected by 18F-FDG-PET.

BAT -: patients without activated BAT on 18F-FDG-PET.

### 18F-FDG-PET/CT

All patients underwent a clinically indicated 18F-FDG-PET/CT on a dedicated scanner (Biograph mCT, Siemens Healthcare, Erlangen, Germany). PET acquisition was performed 60±3 min after injection of 326±19 MBq 18F-FDG. Patients were positioned with elevated arms within the PET/CT scanner in order to optimize CT image quality and dose exposure of the torso. Activated BAT was defined on PET/CT as areas within the supraclavicular adipose tissue compartments with focal uptake of 18F-FDG.

**Fig 1:**
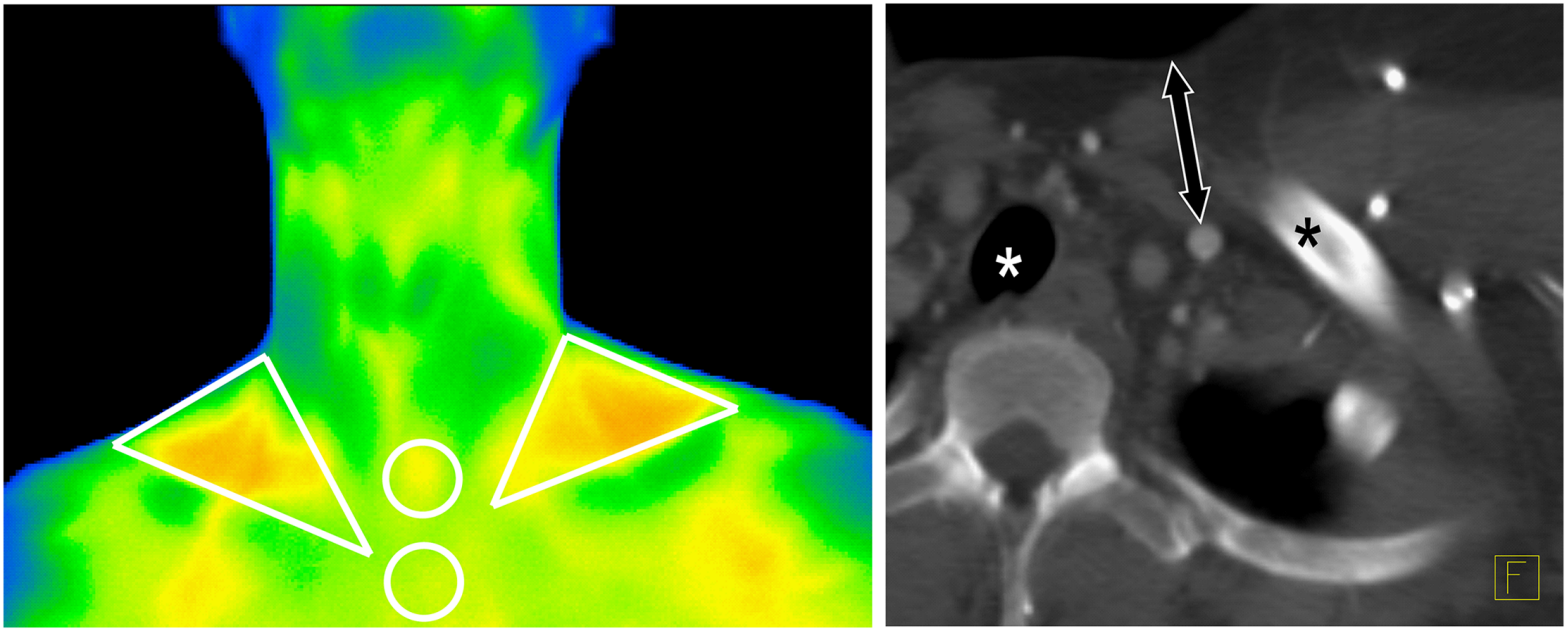
Illustration of performed measurements. Left (thermographic data): Supraclavicular ROIs were of triangular shape and placed within the lateral neck triangle that is formed by the clavicle, the sternocleidomastoid muscle and the lateral neck contour. Jugular and presternal ROIs were circular. Right (CT data): As a measure for SCAT thickness of the supraclavicular regions, the minimal distance between the vascular compartment of the neck and the skin surface was measured (double arrow). Asterisks mark the left clavicle and the trachea.

Contrast-enhanced or non-enhanced CT was performed depending on the clinical question. Local thickness of subcutaneous adipose tissue (SCAT) of the supraclavicular region was measured on transverse CT clices as the minimal distance between the vascular compartment of the neck and the skin surface (Fig 1).

### Infrared thermography

Infrared thermography was performed immediately prior to FDG injection using a high resolution infrared camera (Varioscan 3021 ST, Jenoptik, Jena, Germany). To this end patients were seated in an upright position and were asked to expose their neck and upper chest regions. The room temperature of the PET suite was kept constant at 21°C at all times. At least 15 minutes prior to thermographic measurements, patients were dressed in a hospital coat and remained seated in order to avoid variations due to physical activity or different clothing. A single static thermographic image (matrix size 360 x 240) was acquired from a distance of 1 meter. Thermometric images were analyzed using the vendor-provided software. Triangular regions of interest (ROIs) were placed in the left and right supraclavicular areas; circular control ROIs were placed over the sternal and jugular regions. Details of ROI placement are described in Fig 1. Mean and maximum skin temperatures of these ROIs were extracted for further analysis.

### Statistical analysis

Data are expressed as mean ± SD. Statistical correlations between metric variables are described using Pearson’s correlation coefficients. Differences in local skin temperature between subjects with and without active BAT were statistically compared using a two-sided unpaired t-test. A linear regression analysis was performed in order to assess the predictive value of the possibly influencing factors BAT activity, age, sex and BMI on mean supraclavicular skin temperature. P-values < 0.05 were considered statistically significant.

## Results

Activated BAT of the supraclavicular adipose tissue compartments was detected in 8.8% of the patients (9/102; 5 female, 4 male). Characteristics of patients with and without activated BAT are summarized in Table 1. As expected, patients with activated BAT were in average significantly younger (p<0.001) and had a significantly lower BMI (P = 0.01) compared to patients without activated BAT.

**Fig 2:**
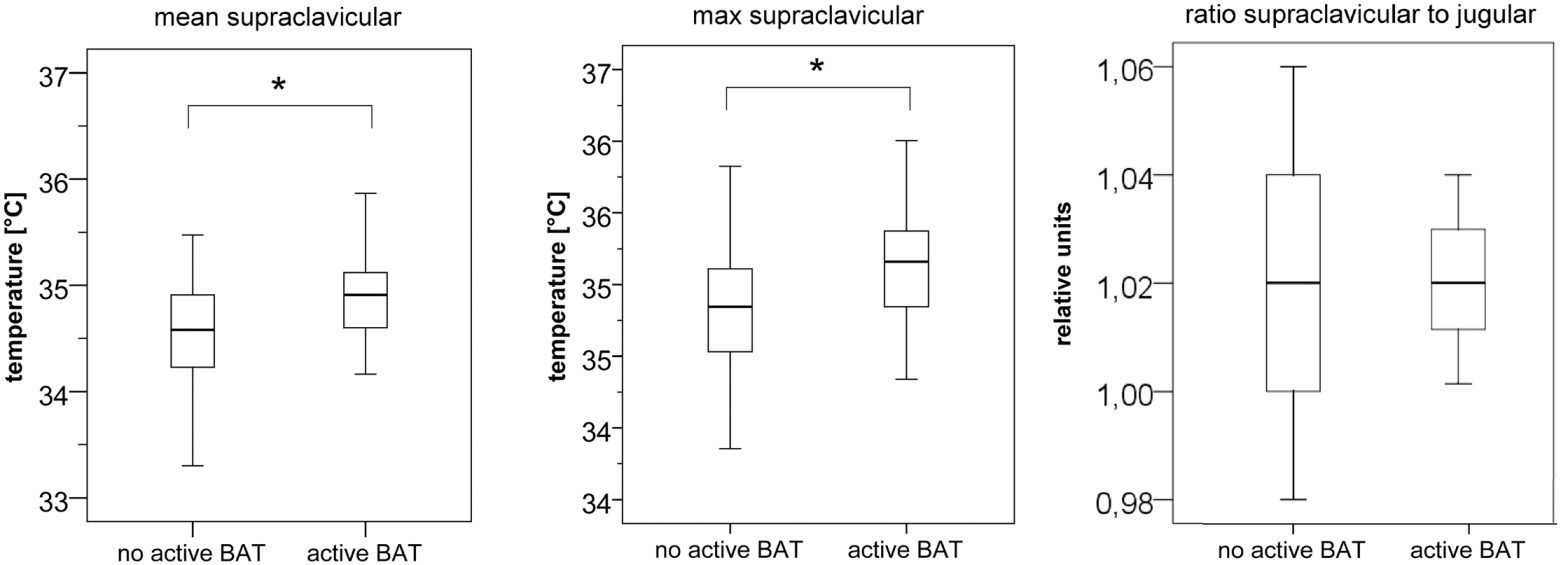
Mean (left) and maximum (middle) skin temperature of the supraclavicular regions in patients without and with active brown adipose tissue. Mean and maximum supraclavicular skin temperatures were significantly different between patients with and without active BAT (* indicates statistically significant differences). No significant difference was observed between BAT-positive and BAT-negative patients when mean supraclavicular skin temperature was normalized relative to the jugular skin temperature (right).

Patients with activated BAT had significantly higher mean and maximum skin temperature over the supraclavicular region compared to patients without activated BAT (mean temperature [°C]: 35±0.5 vs. 34.6±0.5, p = 0.02; maximum temperature [°C]: 35.2±0.6 vs. 34.8±0.5, p = 0.03; Fig 2). Normalization of mean supraclavicular skin temperature relative to mean jugular temperature did not allow for a better differentiation of patients with and without activated BAT (Fig 2). No significant difference in mean skin temperature was observed in the presternal (p = 0.16) or jugular (p = 0.39) regions between patients with and without activated BAT.

**Fig 3:**
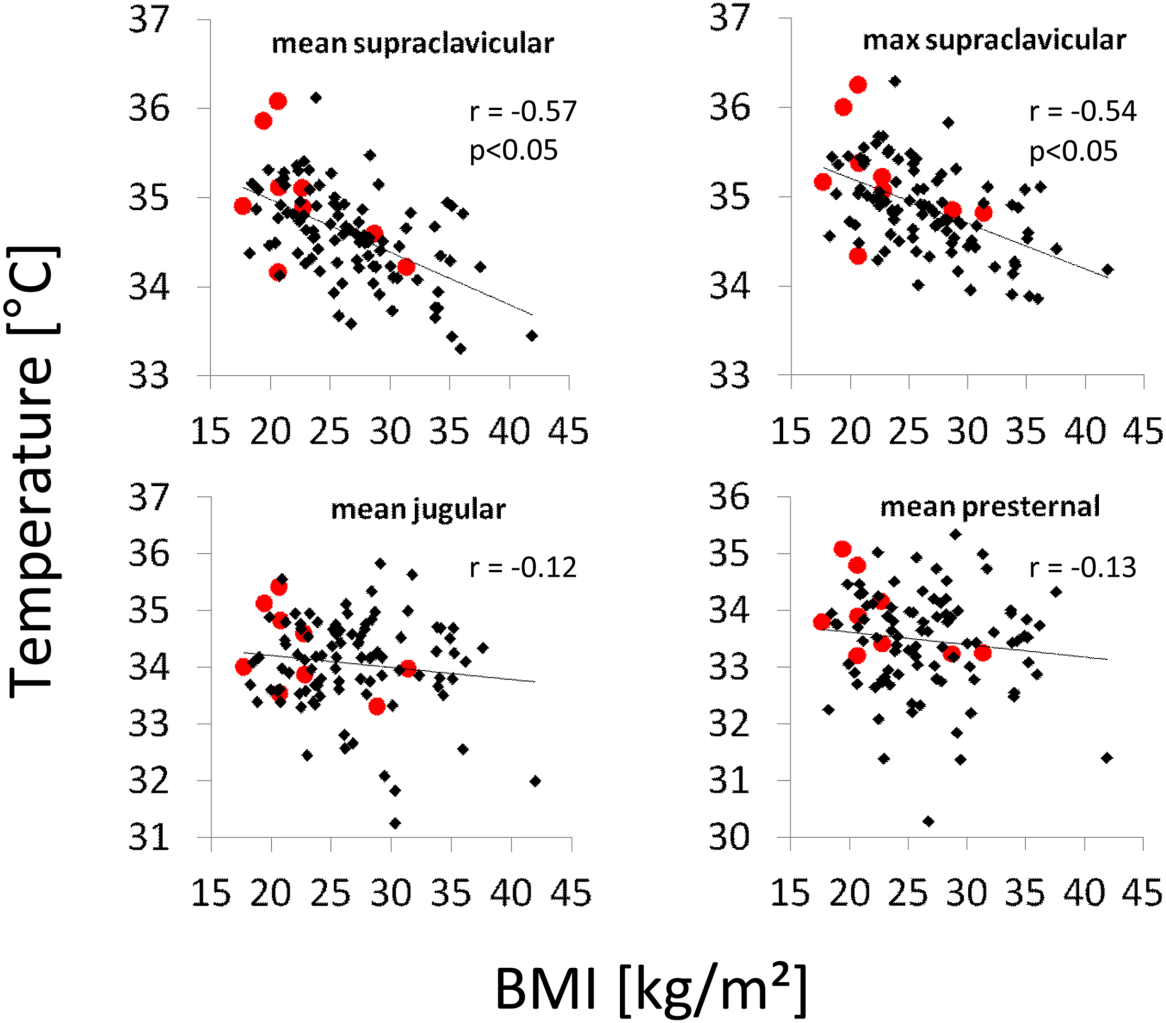
Correlation of skin temperatures of the supraclavicular, the presternal and the jugular regions with body mass index. Red dots mark patients with activated BAT in FDG-PET. r denotes Pearson’s correlation coefficients.

A significant negative correlation was observed between mean and maximum skin temperature of the supraclavicular regions and the patient body mass index (r = -0.57; p<0.01 and r = -0.54; p<0.01 respectively; Fig 3). No significant correlation could be observed between BMI and skin temperature of the jugular and presternal regions (r = -0.12; p = 0.21 and r = -0.13; p = 0.19 respectively; Fig 3).

**Fig 4:**
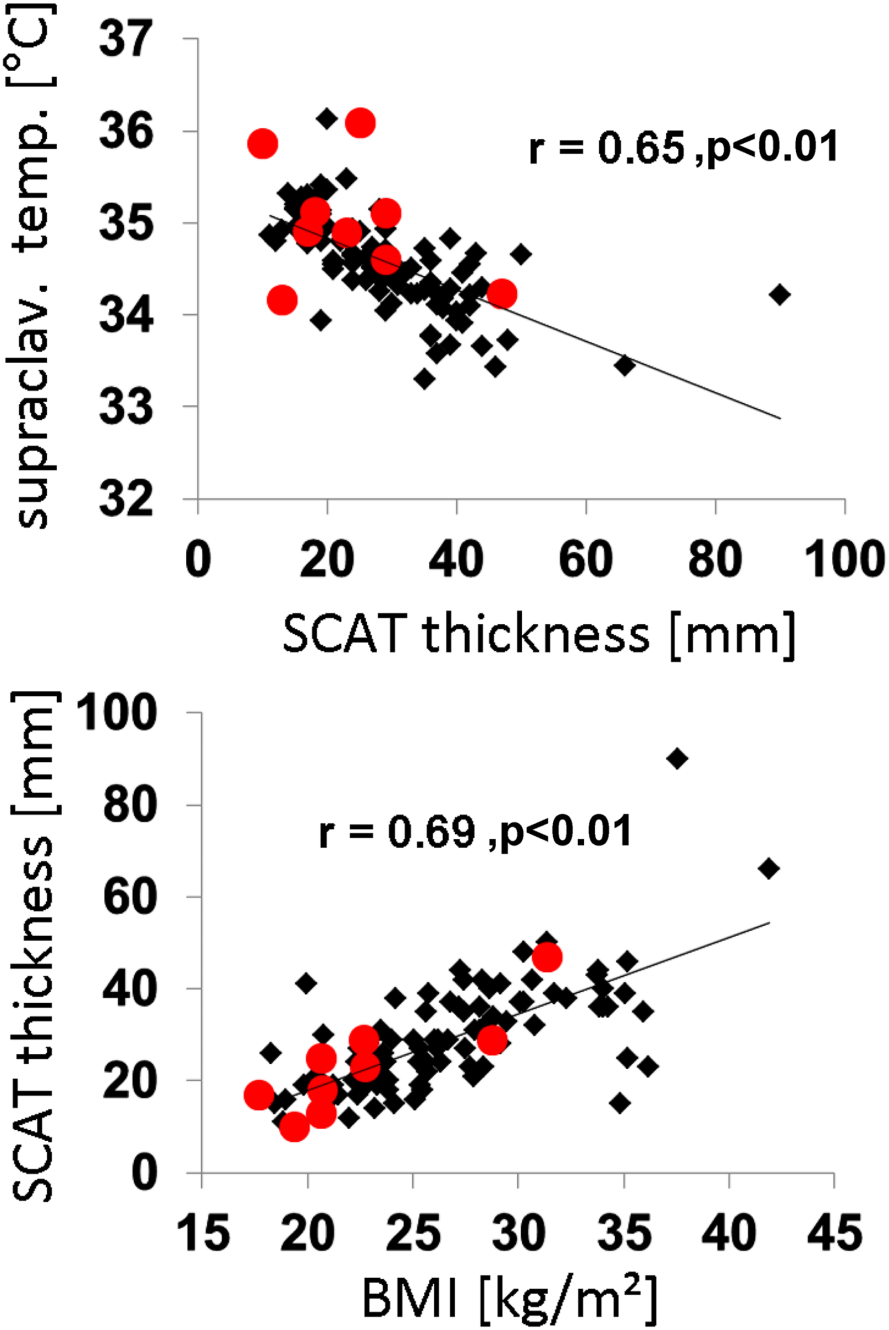
Correlation between supraclavicular skin temperature and SCAT thickness (top) and between BMI and supraclavicular SCAT thickness (bottom). r denotes Pearson’s correlation coefficient.

A significantly negative correlation was observed between mean supraclavicular skin temperature and local thickness of SCAT (r = -0.65, p<0.01, Fig 4). Local thickness of SCAT correlated positively with BMI (r = 0.69, p < 0.01, Fig 4).

Linear regression analysis revealed a significant predictive value of BMI on mean supraclavicular skin temperature (p < 0.01). The presence of active BAT (p = 0.22), patient age (p = 0.81) and patient sex (p = 0.07) however had no significant predictive meaning.

**Fig 5:**
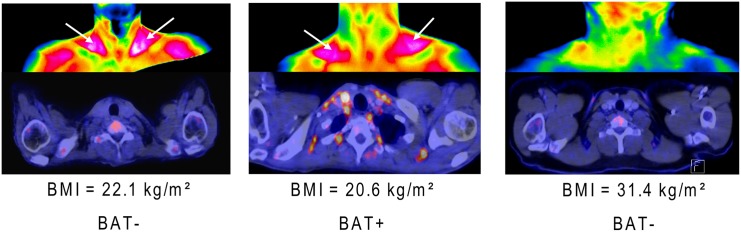
Examples illustrating the study results. Thermographic images (top) and PET images (bottom) of patients without (left and right) and a patient with (middle) metabolically active BAT. Patients with low BMI (left and middle) display relatively high skin temperatures of the supraclavicular regions (arrows) independent of the presence of activated BAT.

Fig 5 shows examples of thermographic as well as PET images of patients with and without activated BAT illustrating the study results.

## Discussion

In this study we aimed to evaluate the feasibility to detect activated BAT by single-time-point infrared thermography under thermoneutral conditions. We detected active BAT by 18F-FDG-PET in about 9% of our study population, which is in good concordance with previously reported prevalences under thermoneutral conditions [22]. We observed significantly higher skin temperature in the supraclavicular regions in patients with activated BAT compared to patients without activated BAT. This result is in concordance with earlier studies that suggested the feasibility of BAT detection using infrared thermography [17,18,20].

However, after correction of our data for the influence of BMI, no statistical effect of activated BAT on skin temperature of the supraclavicular region could be observed. In contrast, supraclavicular skin temperature was negatively correlated with BMI. These results suggest that variations in supraclavicular SCAT thickness between individuals strongly influence supraclavicular skin temperature. Higher skin temperature in the supraclavicular region in patients with low BMI can be mainly explained by a relatively thin layer of isolating adipose tissue. Most likely, the relatively increased supraclavicular skin temperature in lean individuals is caused by large blood vessels that are located in this anatomic area, mainly the subclavian and carotid arteries. These vessels convey thermal energy from the body core to the extremities which might result in an improved detection in lean compared to obese individuals. This conclusion is supported by the observation that skin temperatures over the supraclavicular region showed a significant negative correlation with local thickness of subcutaneous adipose tissue.

Recent studies on the feasibility of detecting BAT under cold exposure reported an increase in supraclavicular skin temperature after cold exposure corresponding to BAT activation [20]. In these studies, the *intra*-individual change in skin temperature was the main effect indicating activation of BAT. In this setting, detection of BAT using dynamic thermography may allow for BAT detection in individuals of different BMI, as long as the dynamic change in temperature is detectable. Under thermoneutral conditions however, when single-time-point thermometry is used, variations in BMI make the specific detection of activated BAT difficult as observed in our study.

The present study has limitations. Due to the experimental setup without dedicated cold exposure, the total number of individuals with active BAT was relatively low. Previous studies have shown that cold exposure is a reliable trigger of BAT activation in humans [2–4]. In this present study however we refrained from using additional cold exposure in order to study the feasibility of detecting BAT under thermoneutral conditions in a setting with low pre-test probability. Furthermore, we were concerned to avoid possible impairment of diagnostic PET quality in the patient population of our study due to deliberately activated BAT. Future studies in healthy volunteers will be possible without this restriction possibly resulting in a better understanding of the association between BAT activation and thermal skin changes under thermoneutral conditions and cold exposure.

In the study setup used in this study an activation of BAT might have happened between infrared thermographic and PET imaging for some patients. However, we minimized the probability of such occurrences by minimizing the time between infrared thermography and FDG injection and by holding the environmental temperature constant between the scans. Our results do not exclude that small changes in local skin temperature can be induced by metabolically active BAT also under thermoneutral conditions. However, alternative thermographic methods would be necessary to reliably detect these changes if present. For example, dynamic thermography under cold exposure and near-infrared spectroscopy were described recently for the detection of active BAT [20,23]. Alternatively, MRI may enable non-invasive temperature measurement of tissue temperature as could be shown in mice [24]. Further studies taking into account possible confounding factors, in particular BMI, are necessary in order to evaluate these possibilities.

## Conclusion

We conclude that supraclavicular SCAT thickness influences supraclavicular skin temperature and thus makes a specific detection of activated BAT using single-time-point thermography difficult. Further studies are necessary to evaluate the possibility of BAT detection under thermoneutral conditions using alternative thermographic methods, e.g. dynamic thermography or MR-based thermometry taking into account BMI as a confounding factor.
